# Lethal Zika Virus Disease Models in Young and Older Interferon α/β Receptor Knock Out Mice

**DOI:** 10.3389/fcimb.2018.00117

**Published:** 2018-04-11

**Authors:** Andrea Marzi, Jackson Emanuel, Julie Callison, Kristin L. McNally, Nicolette Arndt, Spencer Chadinha, Cynthia Martellaro, Rebecca Rosenke, Dana P. Scott, David Safronetz, Stephen S. Whitehead, Sonja M. Best, Heinz Feldmann

**Affiliations:** ^1^Laboratory of Virology, Rocky Mountain Laboratories, Division of Intramural Research, National Institute of Allergy and Infectious Diseases, National Institutes of Health, Hamilton, MT, United States; ^2^Rocky Mountain Veterinary Branch, Division of Intramural Research, National Institute of Allergy and Infectious Diseases, National Institutes of Health, Hamilton, MT, United States; ^3^Zoonotic Diseases and Special Pathogens, National Microbiology Laboratory, Public Health Agency of Canada, Winnipeg, MB, Canada; ^4^Laboratory of Infectious Diseases, Division of Intramural Research, National Institute of Allergy and Infectious Diseases, National Institutes of Health, Bethesda, MD, United States

**Keywords:** Zika virus, strains, pathogenesis, animal model, IFNAR^−/−^ mice, uniform lethality, countermeasures

## Abstract

The common small animal disease models for Zika virus (ZIKV) are mice lacking the interferon responses, but infection of interferon receptor α/β knock out (IFNAR^−/−^) mice is not uniformly lethal particularly in older animals. Here we sought to advance this model in regard to lethality for future countermeasure efficacy testing against more recent ZIKV strains from the Asian lineage, preferably the American sublineage. We first infected IFNAR^−/−^ mice subcutaneously with the contemporary ZIKV-Paraiba strain resulting in predominantly neurological disease with ~50% lethality. Infection with ZIKV-Paraiba by different routes established a uniformly lethal model only in young mice (4-week old) upon intraperitoneal infection. However, intraperitoneal inoculation of ZIKV-French Polynesia resulted in uniform lethality in older IFNAR^−/−^ mice (10–12-weeks old). In conclusion, we have established uniformly lethal mouse disease models for efficacy testing of antivirals and vaccines against recent ZIKV strains representing the Asian lineage.

## Introduction

Zika virus (ZIKV) is a human pathogen that was first isolated in 1947 from a sentinel rhesus monkey in the Zika forest of Uganda (Dick, 1952; Dick et al., 1952). The virus is readily borne by *Aedes aegypti* mosquitos and propagates in vertebrate hosts (Hayes, 2009; Faye et al., 2014). In humans, as many as 80% of ZIKV infections are believed to be asymptomatic (Duffy et al., 2009). Symptomatic cases typically manifest as Zika fever, a mild illness characterized by headache, maculopapular rash, myalgia, and conjunctivitis (Bearcroft, 1956; Simpson, 1964). For many decades, Zika fever was considered a rare illness, occurring only sporadically in humans. In recent years, several major outbreaks, including the outbreak in French Polynesia and the ongoing outbreak in the Americas, have been associated with severe complications, such as Guillain-Barré syndrome (Cao-Lormeau et al., 2016). Additionally, sexual transmission of the virus has been documented (Musso et al., 2015; Frank et al., 2016), and there is now a scientific consensus that ZIKV infection in pregnant mothers increases the risk of giving birth to a child with a spectrum of neurological disorders, of which the most severe is microcephaly (Mlakar et al., 2016). Considering these developments, the World Health Organization previously declared the New World ZIKV epidemic to be a Public Health Emergency of International Concern, with ZIKV now representing an enduring public health challenge.

ZIKV is a member of the Spondweni virus clade in the *Flavivirus* genus. It shares many structural similarities with other pathogenic flaviviruses, such as Dengue virus (DENV), Japanese encephalitis virus (JEV), and West Nile virus (WNV) (Kuno and Chang, 2007; Kostyuchenko et al., 2016). The ZIKV genome comprises a positive-sense, single-stranded RNA of about 11-kb in length, which is expressed as a single polyprotein. This polyprotein is cleaved by viral and host proteases into 10 functional polypeptides, three structural proteins (capsid [C], pre-membrane [prM], envelope [E]) and seven non-structural proteins (NS1, NS2A, NS2B, NS3, NS4A, NS4B, and NS5) (Kuno and Chang, 2007; Kostyuchenko et al., 2016). There are two distinct lineages of ZIKV (Figure S1): the ancestral African lineage from which the 1947 isolate (MR766) was cultivated and the emergent Asian lineage responsible for the present epidemic (Faye et al., 2014; Zanluca et al., 2015). Although these lineages are genetically distinct, ZIKV was shown to have only one serotype (Dowd et al., 2016). Recently, changes in the NS1 protein were reported to determine ZIKV infectivity in *Aedes aegypti* mosquitos (Liu et al., 2017), yet it is still unknown to what extent genetic differences between the strains affect disease severity in humans.

The most amenable small animal models for ZIKV infection are mice lacking interferon responses recapitulating certain features of human disease like meningoencephalitis and fetal brain malformations (Miner and Diamond, 2017; Morrison and Diamond, 2017). For ZIKV countermeasure development we favored the C57BL/6 interferon receptor α/β knock out (IFNAR^−/−^) mouse model as these mice, despite being immunocompromised, can still develop protective immune responses (Hinkula et al., 2017). However, IFNAR^−/−^ mice, particularly older animals, do not display uniform lethality upon ZIKV infection, which is not ideal when evaluating the efficacy of a vaccine because it takes 4–8 weeks to develop protective immune responses. Therefore, we optimized this IFNAR^−/−^ mouse model utilizing recent ZIKV strains from the American sublineage. We found age- and inoculation route-dependent uniform lethality with ZIKV-Paraiba causing fatal infections in young (4-week old) and ZIKV-French Polynesia in older (10–12-weeks old) IFNAR^−/−^ mice. Thus, we were able to establish age-dependent uniformly lethal IFNAR^−/−^ mouse models suitable for countermeasure efficacy testing against recent ZIKV strains.

## Materials and methods

### Animal ethics and safety statements

Research was approved by the NIAID/RML Institutional Animal Care and Use Committee (IACUC) and conducted in compliance with all necessary guidelines and regulations. Our research facility is fully accredited by the Association for the Assessment and Accreditation of Laboratory Animal Care International (AAALAC) and has an approved Office of Laboratory Animal Welfare (OLAW) Assurance number (#A4149-01). All procedures were conducted by trained personnel under the supervision of veterinarians, and all invasive clinical procedures were performed while animals were anesthetized. Humane endpoint criteria, as specified by the IACUC approved scoring parameters, were used to determine when animals should be humanely euthanized. ZIKV is classified as biosafety level 2 (BSL2) pathogen and all work with the ZIKV isolates was approved by the Institutional Biosafety Committee (IBC) under BSL2 conditions.

### ZIKV stock propagation

The following ZIKV isolates were used: ZIKV-Paraiba (human, Brazil 2015; GenBank accession number KX280026.1) (Tsetsarkin et al., 2016), ZIKV-French Polynesia (human, 2013) (Fonseca et al., 2014), and ZIKV-MR766 (rhesus macaque, Uganda 1947; BEI Resources NR-50065) (Dick et al., 1952). For ZIKV propagation, C6/36 mosquito cells were grown in MEM, 10% heat inactivated FBS, 2 mM Glutamine, 1X non-essential amino acids and Pen Strep (all reagents from Gibco/Life Technologies). C6/36 cells were plated at 13-15 x 10^6^ cells/p100 dish and incubated overnight at 32°C, 5% CO_2_. The next day, media was aspirated from the dish and 5 ml of complete media containing ZIKV at a final MOI = 0.01 was added for 1 h with periodic rocking at 32°C. After 1 h, 10 ml of complete media was added to each p100 plate and the cells were incubated for 72 h. Cell supernatant was pooled from multiple plates and centrifuged at 1,500 RPM for 3 min. Supernatant was aliquoted, flash frozen on dry ice and stored at −80°C.

### Titration of ZIKV stocks by plaque assay

VeroE6 cells were plated at 2 × 10^5^ cells/24 well in DMEM, 10% FBS, Pen Strep (Life Technologies) and incubated overnight at 37°C. The next day, 10-fold serial dilutions of the ZIKV stock were prepared in complete DMEM. Cells were infected with 125 ul of each dilution to be tested in duplicate at 37°C for 1 h. Virus was aspirated and wells were washed 1X with PBS. Cells were overlayed with 1 ml of 1.5% carboxymethylcellulose (CMC) (Sigma) dissolved in MEM and incubated at 37°C for 4–5 days. For fixation, 1 ml of 10% formalin was added directly on top of the CMC and plates were incubated at room temperature overnight. Wells were washed 3–4 times with water to remove the CMC and 1% crystal violet was added to each well for 15–30 min at room temperature to visualize plaques. Wells were washed with water, plaques were counted and titer was calculated.

### ZIKV RT-qPCR

Total RNA was extracted from tissues using the RNeasy Mini Kit (Qiagen) according to manufacturer's directions. Total RNA was extracted from whole blood using the QIAamp Viral RNA Mini Kit (Qiagen) according to manufacturer's directions. ZIKV-specific RNA was detected using the primers and probe set described by Faye and colleagues (Faye et al., 2013). All RT-qPCR reactions were performed on the Rotor-Gene 6000 thermal cycler (Qiagen) using the QuantiFast Probe RT-PCR Kit (Qiagen) according to manufactuer's instructions.

### *In vitro* growth kinetics

C6/36 cells were seeded at 2 × 10^6^ cells/6 well in MEM, 10% heat inactivated FBS, 2 mM Glutamine, 1X non-essential amino acids and Pen Strep. VeroE6 cells were plated at 5 × 10^5^ cells/6 well in DMEM, 10% FBS and Pen Strep. Both cell lines were incubated overnight to achieve ~90% confluency the next day. Cells were infected with 0.5 ml ZIKV at an MOI of 0.01 in triplicate. After 1 h, 3 ml of DMEM, 2% FBS, Pen/Strep and L-glutamine were added to each well. At time points 0, 12, 24, 36, 48, 60, 72, and 96 h after infection, 1 ml of supernatant was removed from the cells and stored at −80°C until titration. After sample removal 1 ml of fresh DMEM, 2% FBS, Pen/Strep and L-glutamine was added to each well.

### Titration of ZIKV samples by TCID_50_ assay

VeroE6 cells were seeded the day before titration in 96-well plates. Frozen tissue samples were thawed, homogenized in DMEM, cleared from debris by centrifugation and 10-fold serial dilutions were prepared. Frozen blood and cell culture supernatants were thawed and 10-fold serial dilutions were prepared. A confluent layer of Vero E6 cells was infected in triplicates per dilution for 1 h at 37°C, then DMEM/2% FBS were added. After 3 days, the plates were analyzed for ZIKV specific cytopathic effect (CPE) and the median tissue culture infectious doses (TCID_50_) were calculated using the Reed-Muench method (Reed and Muench, 1938).

### *In vivo* mouse studies

Female and male C57BL/6 interferon receptor α/β knock out (IFNAR^−/−^) mice ranging from 4–12 weeks of age were inoculated with the indicated doses of ZIKV (diluted in sterile DMEM without supplements) by either subcutaneous (between the shoulder blades) or intraperitoneal injection in a total volume of 100 μl. Additionally, footpad (left rear foot) inoculation was performed with a 50 ul inoculum per mouse. The animals were monitored for signs of illness and weighed daily. On necropsy days, mice were anesthetized, bled, euthanized and tissue samples were taken and stored at −80°C until titration. Surviving mice were euthanized 28 days after infection.

### Pathology and *in situ* hybridization

Tissues were collected and fixed in 10% Neutral Buffered Formalin for a minimum of 7 days. Tissues were placed in cassettes and processed with a Sakura VIP-7 Tissue Tek on a 12 h automated schedule using a graded series of ethanol, xylene, and Ultraffin. Embedded tissues were sectioned at 5 μm and dried overnight at 42°C prior to staining with hematoxylin and eosin (H&E). Tissue samples were evaluated in detail and the following scoring system was applied: 0 = no lesions; 1 = small number of necrotic cells; 2 = moderate necrosis; 3 = significant necrosis; 4 = coalescing necrosis; 5 = diffuse necrosis. Detection of ZIKV RNA was performed using the RNAscope 2.5 VS assay (Advanced Cell Diagnostics Inc.) on the Ventana Discovery ULTRA as previously described (Wang et al., 2012) and in accordance with the manufacturer's instructions. Briefly, tissue sections were deparaffinized and pretreated with heat and protease before hybridization with V-ZIKA-pp-02 probe for ZIKV (ACDBio). Peptidylprolyl isomerase B (Ppib) and the bacterial gene, *dapB*, were used as positive and negative controls, respectively.

### Phylogenetic analysis

The polyprotein coding regions of 65 flavivirus strains were obtained from NCBI, including isolates of ZIKV, DENV, JEV, and WNV. A multiple sequence alignment based on fast Fourier transform (MAFFT) was performed (Katoh et al., 2002). The alignment was then used to construct an unrooted neighbor-joining tree using the Tamura-Nei genetic distance model. Clades were identified on the basis of the geographical origins of the isolates.

### Statistical analyses

All statistical analysis was performed in Prism 7 (GraphPad). All survival curves were compared with the Log-rank (Mantel Cox) survival test. Body weight curves were analyzed using parametric *t*-tests. Growth kinetics were analyzed using one-way ANOVA with Tukey multiple comparison post-test.

## Results

**Figure 1:**
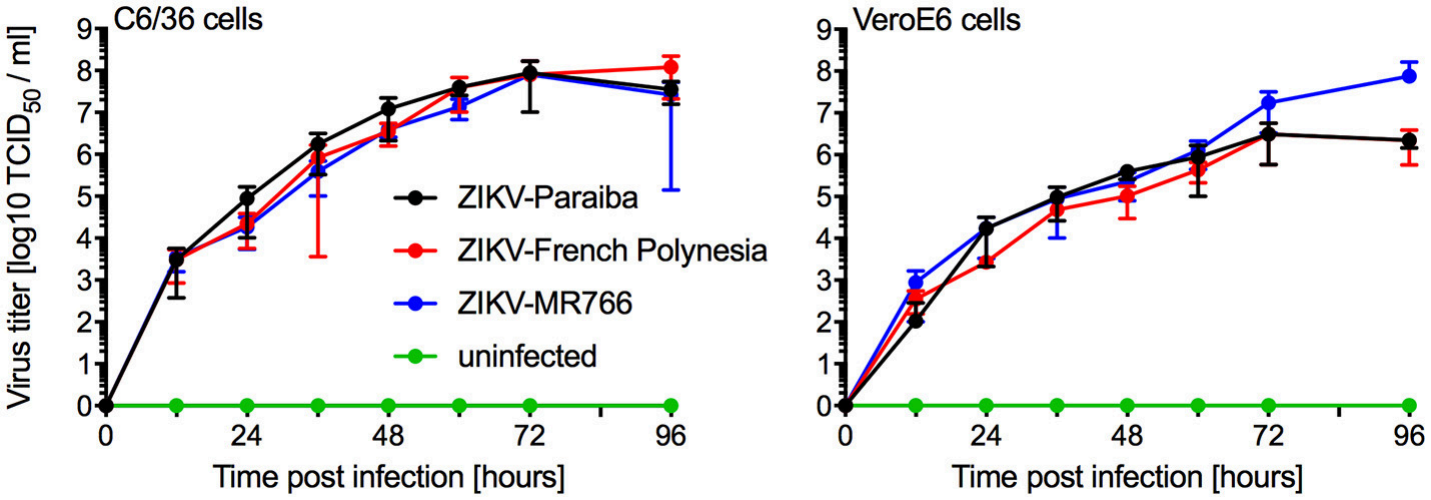
*In vitro* growth kinetics. C6/36 and VeroE6 cells were infected in triplicates with three different ZIKV isolates at an MOI of 0.01 or remained uninfected. Supernatant samples were taken at the indicated time points and were stored at −80°C until titration on VeroE6 cells. One representative experiment is shown, error bars indicate standard deviation.

We compared *in vitro* growth kinetics of three different ZIKV strains representing the African and Asian lineage as well as the American sublineage (Figure S1). Replication kinetics of ZIKV-MR766 (rhesus macaque, Uganda 1947) (Dick et al., 1952), ZIKV-French Polynesia (human, 2013) (Fonseca et al., 2014), and ZIKV-Paraiba (human, Brazil 2015) (Tsetsarkin et al., 2016) were evaluated on mosquito (C6/36) and mammalian (VeroE6) cells. Cells were infected in triplicate at a multiplicity of infection (MOI) of 0.01 and samples were taken at the indicated time points. The three ZIKV isolates showed similar kinetics and grew to similar endpoint titers demonstrating little difference between isolates representing the different clades (Figure 1). Notably, ZIKV-MR766 grew to a slightly higher, albeit not significantly different endpoint titer on VeroE6 cells, which is likely due to the extensive passaging history of this isolate in mouse brains (Dick, 1952; Dick et al., 1952). However, for all mouse infection experiments ZIKV stocks propagated in C6/36 cells were used to more closely mimic the natural transmission of ZIKV through mosquitos.

**Figure 2:**
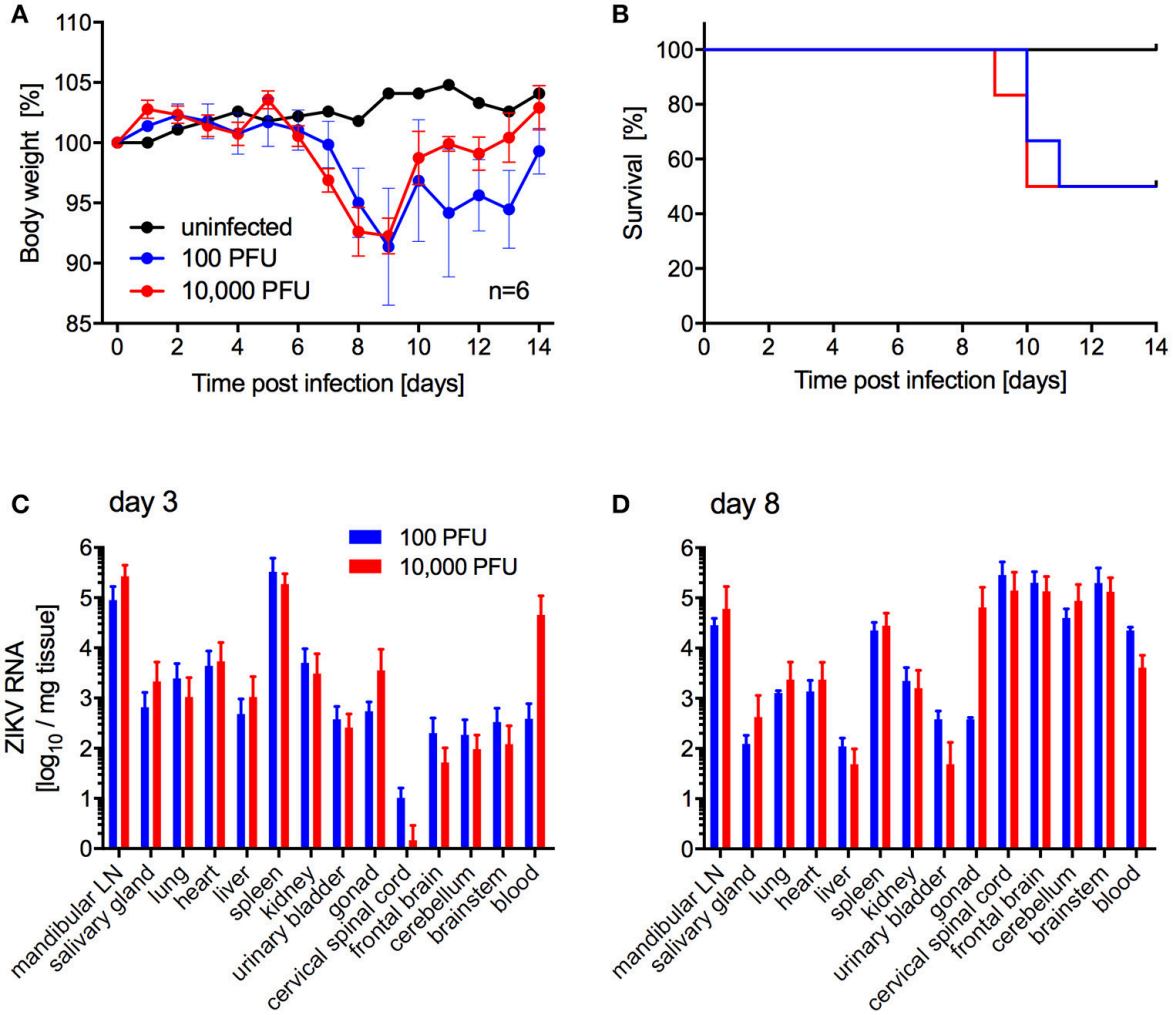
ZIKV-Paraiba infection in IFNAR^−/−^ mice. Female and male mice (5–8-weeks old) were infected with 100 PFU (*n* = 12) or 10,000 PFU (*n* = 13) of ZIKV-Paraiba or remained uninfected (*n* = 1). Body weight changes **(A)** and survival curves **(B)** for uninfected (*n* = 1) and a subset of ZIKV-infected mice (*n* = 6 per dose) are shown. On day 3 after infection, 3 mice in both groups were euthanized for virus load determination in blood and tissues **(C)**. On day 8 after infection, ZIKV virus loads were determined in blood and tissues for the 100 PFU (*n* = 3) and 10,000 PFU (*n* = 5) groups **(D)**. Error bars indicate standard deviation. Differences in survival, body weights loss and viral loads for the two doses were not statistically significant.

**Figure 3:**
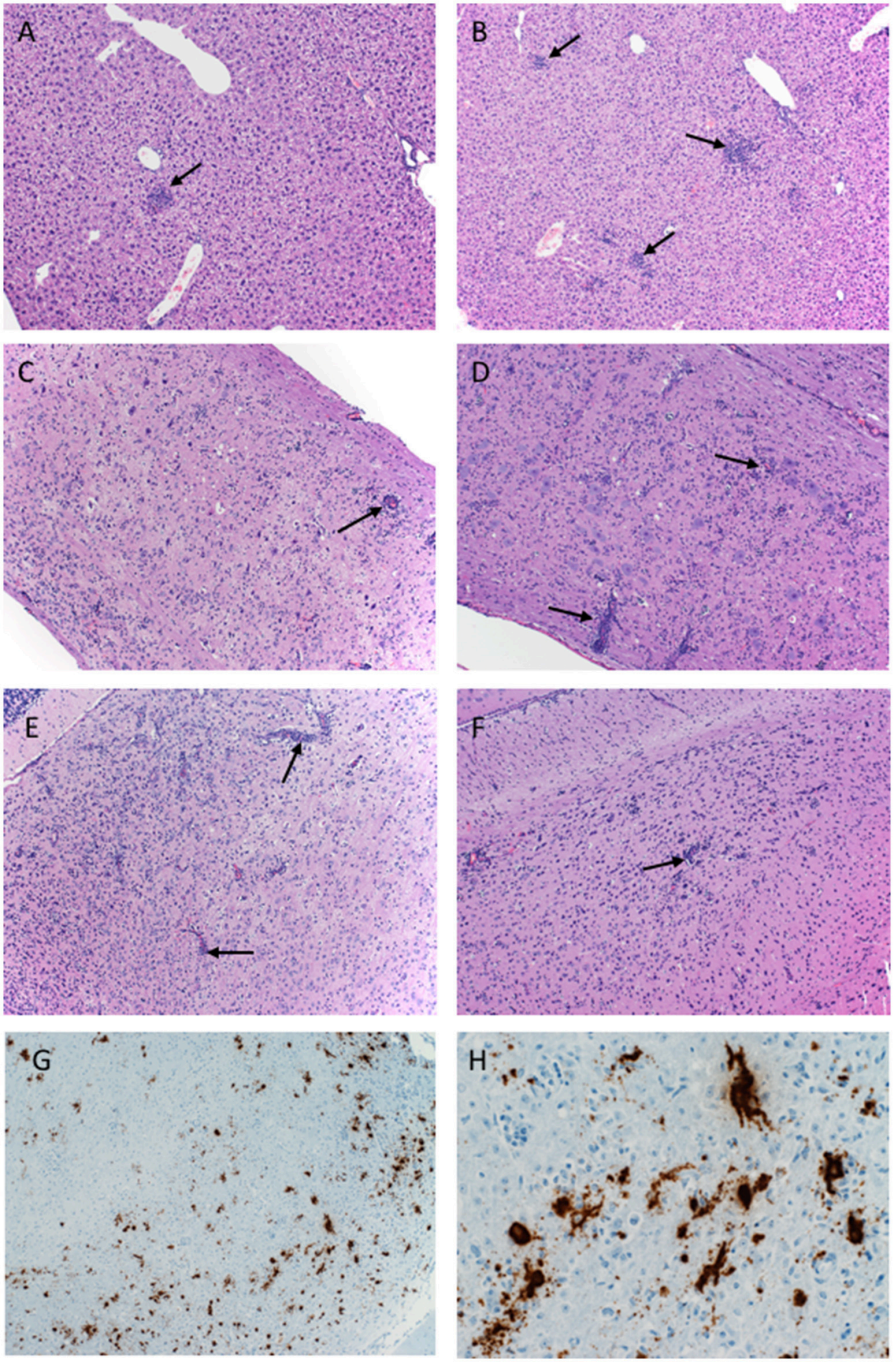
Pathology of ZIKV-Paraiba infection in IFNAR^−/−^ mice. Tissue samples collected on day 8 after infection were fixed in formalin and stained with hematoxylin and eosin. **(A)** Low dose infection (100 PFU): liver 10x; arrows indicate hepatic inflammation. **(B)** High dose infection (10,000 PFU): liver 10x; arrows indicate hepatic inflammation. **(C)** Low dose infection (100 PFU): spinal cord 10x; arrows indicate vascular cuffing. **(D)** High dose infection (10,000 PFU): spinal cord 10x; arrows indicate vascular cuffing. **(E)** Low dose infection (100 PFU): brain 10x; arrows indicate vascular cuffing. **(F)** High dose infection (10,000 PFU): brain 10x; arrows indicate vascular cuffing. ZIKV RNA was also detected by *in-situ* hybridization in the brain, **(G)** 10x, **(H)** 40x. x = magnification.

Next, we investigated the potential of a human isolate from Brazil 2015 associated with a microcephaly case, ZIKV-Paraiba, to cause lethal disease in IFNAR^−/−^ mice to establish a model for countermeasure testing as described previously for other ZIKV strains (Lazear et al., 2016; Miner et al., 2016; Tripathi et al., 2017). In the first study, we inoculated 5–8-weeks old IFNAR^−/−^ mice with 100 or 10,000 PFU per animal subcutaneously (s.c.) to more closely mimic natural infection and observed the mice daily for signs of disease including changes in body weight. On days 3 and 8 after infection, a subset of the animals was euthanized to collect blood and tissue samples for virus load determination and pathology. Starting on days 6 and 7 post-infection, the animals lost weight independent of the dose of ZIKV (Figure 2A). Approximately 50% of the mice succumbed to disease or were euthanized between days 9 and 11 due to development of neurological signs such as hind limb paralysis (Figure 2B) with no difference between the inoculation doses. Similarly, there was no significant difference between ZIKV RNA loads in the tissues collected on day 3 (Figure 2C) and day 8 (Figure 2D). However, ZIKV could only be isolated from the samples with the highest RNA levels like mandibular lymph node, spleen (both groups) and blood (10,000 PFU group only) (data not shown). By day 8, ZIKV RNA was detected in the gonad, spinal cord and brain (Figure 2D) and a few animals presented with enlarged spleens independent of the inoculation dose. Interestingly, although ZIKV could not be isolated from the liver despite presence of viral RNA (Figure 2D), the mice presented with acute hepatitis including hepatocellular necrosis (Figures 3A,B).

Histologically, mice necropsied on day 3 after infection (*n* = 3 per group) demonstrated no lesions that could be attributed to ZIKV infection (data not shown). However, by day 8 after infection, animals in both groups (low dose *n* = 3; high dose *n* = 5) developed hepatic (Figures 3A,B) and neurologic lesions that were essentially identical (Figures 3C–F). Both low and high dose groups developed mild to moderate subacute hepatitis demonstrated by multiple foci composed of small numbers of lymphocytes, neutrophils and macrophages admixed with necrotic hepatocytes (Figures 3A,B). These mice also developed a mild to marked, multifocal to coalescing, encephalitis characterized by large numbers of necrotic neurons, satellitosis and microgliosis (Figures 3E,F). Multifocally vessels are bounded by small to moderate numbers of lymphocytes (perivascular cuffing) (Figures 3E,F). The spinal cords were similarly affected as there was multifocal to coalescing myelitis with large numbers of necrotic neurons, satellitosis, microgliosis and perivascular cuffing (Figures 3C,D). *In situ hybridization* revealed ZIKV replication primarily in neurons (Figures 3G,H). The lesions caused by ZIKV replication in the brain contributed to the neurological signs the mice developed during disease. Additionally, these mice presented with minimal to mild follicular hyperplasia and minimal extramedullary hematopoiesis in the spleen which is the likely cause of the splenomegaly noted at necropsy. Altogether, s.c. infection of IFNAR^−/−^ mice with ZIKV-Paraiba results in similar viral organ titers and typical disease as described for other ZIKV strains but uniform lethality could not be achieved (Miner and Diamond, 2017; Morrison and Diamond, 2017).

**Figure 4:**
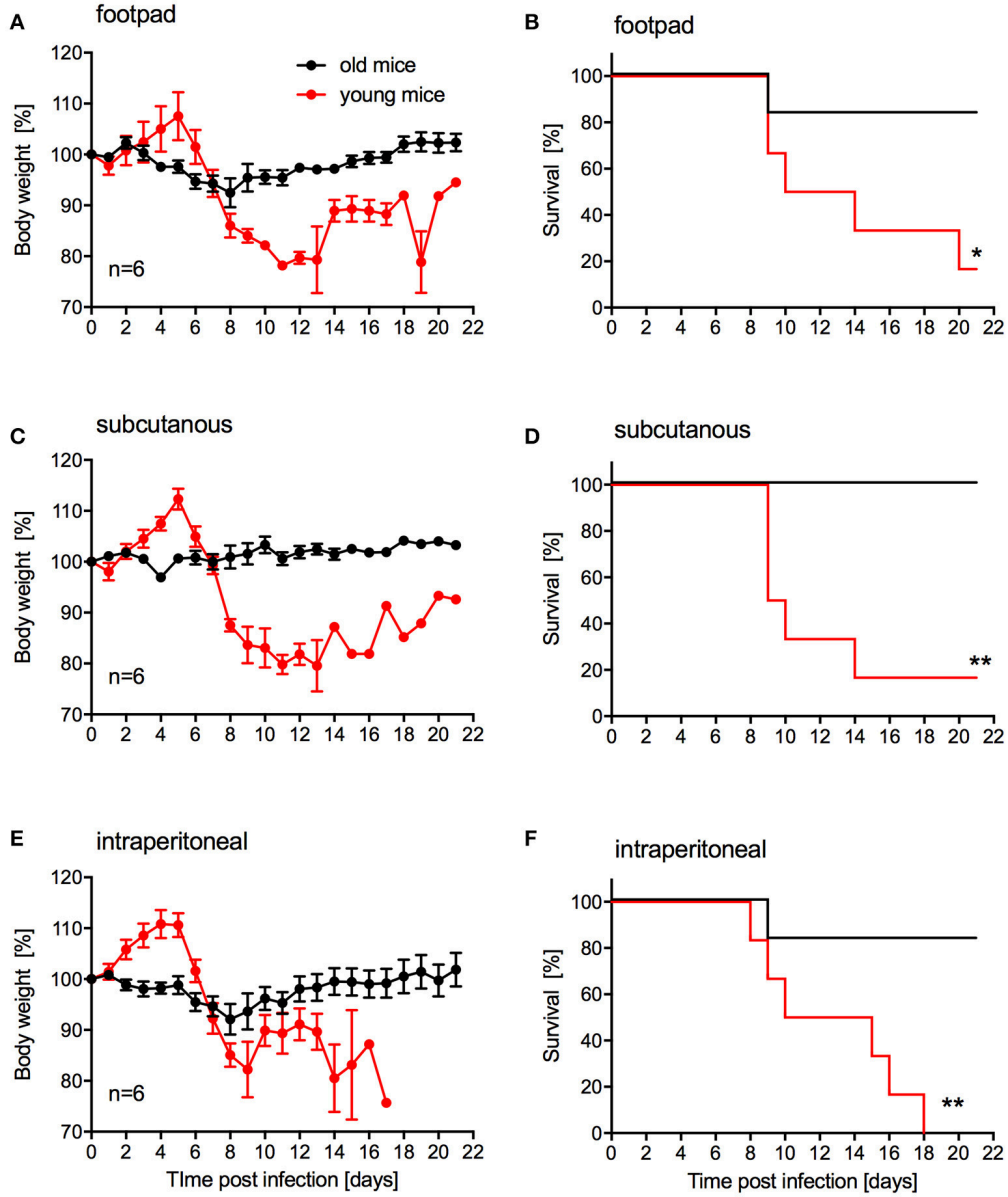
Influence of inoculation route on disease progression in young and older IFNAR^−/−^ mice. Groups of 6 young (4 weeks old) or old (10–12 weeks old) mice, mixed sex, were infected with 100,000 PFU ZIKV-Paraiba via the indicated route. Body weight changes **(A)** and survival **(B)** for footpad inoculation. Body weight changes **(C)** and survival **(D)** for subcutaneous inoculation. Body weight changes **(E)** and survival **(F)** for intraperitoneal inoculation. Error bars indicate standard error of the mean. Statistically significant results are indicated as follows: ^*^p < 0.05 and ^**^p < 0.01.

Our main goal was to develop a lethal mouse model suitable and reliable for ZIKV countermeasure development. For efficacy testing of therapeutic drugs, challenge can be performed in relatively young animals. However, for vaccine efficacy testing challenge will occur in older animals as development of immunity upon vaccination generally takes several weeks. While s.c. inoculation was also used by Dowall *et al*. in their study using A129 mice (Dowall et al., 2016), other groups have established ZIKV mouse models using intraperitoneal (i.p.) or footpad inoculation (Aliota et al., 2016a; Lazear et al., 2016; Rossi et al., 2016). In addition, two other groups have observed phenotypic differences after ZIKV infection of mice with different age (Lazear et al., 2016; Rossi et al., 2016). Thus, we compared the pathogenicity of ZIKV-Paraiba following different inoculation routes in young (4-week old) and older IFNAR^−/−^ mice (10–12-weeks old). We infected groups (mixed gender in each group) of 6 mice with 10^5^ PFU of ZIKV-Paraiba by the footpad, s.c. or i.p. route and documented disease progression. Independent of the route of infection, very few of the older mice demonstrated significant weight loss or other signs of disease (Figures 4A,C,E) except for one mouse in each of the footpad and i.p. infection groups that succumbed to infection (Figures 4B,F). In contrast, almost all the young mice developed significant disease including weight loss after ZIKV-Paraiba infection independent of the route of infection (Figures 4A,C,E). However, only the i.p. inoculation group resulted in uniform lethality for young mice (Figures 4B,D,F). We determined the ZIKV-Paraiba dose causing 50% lethality (LD_50_) as 1 PFU in young mice (Figure S2).

**Figure 5:**
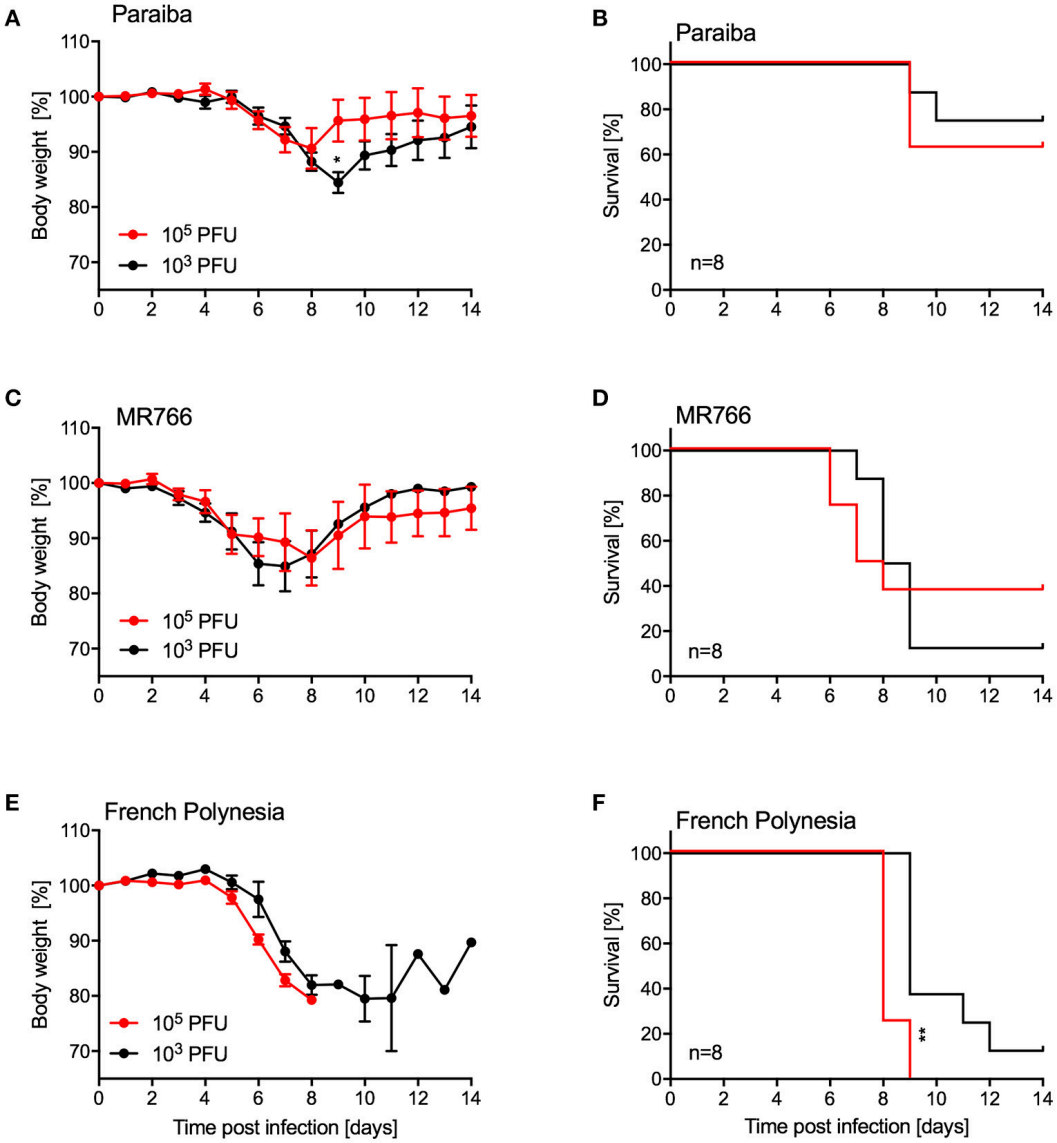
Disease in older IFNAR^−/−^ mice using different ZIKV isolates. Groups of female and male mice (10–12 weeks old; *n* = 8 per group) were intraperitoneally infected with the indicated doses of ZIKV-Paraiba, -MR766, or -French Polynesia. Body weight changes **(A)** and survival **(B)** for ZIKV-Paraiba infection. Body weight changes **(C)** and survival **(D)** for ZIKV-MR766. Body weight changes **(E)** and survival **(F)** for ZIKV-French Polynesia infection. Error bars indicate standard error of the mean. Statistically significant results are indicated as follows: ^*^p < 0.05 and ^**^p < 0.01.

Since we were unable to establish uniform lethality in older IFNAR^−/−^ mice using ZIKV-Paraiba, we infected groups of older mice (10–12-weeks old, 4 females and 4 males per group) with two additional ZIKV strains representing the African and Asian lineages (Figure 1). For this, we used the i.p. installation defined as being the most potent inoculation route for ZIKA-Paraiba (Figure 4). Mice i.p. infected with either 10^3^ or 10^5^ PFU ZIKV-Paraiba showed ~10–15% body weight loss on days 8–10 (Figure 5A) and 75 and 62.5% survived, respectively (Figure 5B). Similar to ZIKV-Paraiba, i.p. infection with 10^3^ or 10^5^ PFU ZIKV-MR766 resulted in ~15–20% body weight loss on days 6–8 (Figure 5C) and 12.5 and 37.5% of the mice survived, respectively (Figure 5D). Surprisingly, infection with the lower dose of this virus resulted in higher but no uniform lethality. Infection (i.p.) with the third isolate, ZIKV-French Polynesia, with 10^3^ or 10^4^ PFU resulted in 87.5 and 100% lethality, respectively (Figure 5F), with the mice showing more serious body weight loss (Figure 5E) compared to the other ZIKV isolates. The one mouse in the 10^3^ PFU group that survived exhibited weight loss but recovered without the development of neurological signs. The LD_50_ in older IFNAR^−/−^ mice was determined to be 4.75 PFU (Figure S3).

## Discussion

The purpose of this study was to develop and characterize uniformly lethal mouse models for future efficacy testing of countermeasures against recent ZIKV strains preferentially representing the American sublineage within the Asian lineage. To this end we compared the pathogenicity of several ZIKV strains representing the distinct lineages and sublineages in IFNAR^−/−^ mice of different age and sex using different routes of infection. We discovered strain-, age-, and inoculation route-dependent differences in ZIKV pathogenicity resulting in two promising age-dependent IFNAR^−/−^ mouse models for efficacy testing of vaccines and therapeutics.

Prior to the animal studies, we wanted to confirm that the ZIKV strains used here did not significantly differ in their capability to grow in mosquito and mammalian cell cultures. ZIKV-Paraiba, ZIKV-French Polynesia, and ZIKV-MR766, representing the American sublineage and the Asian and African lineage, respectively, did not result in distinct *in vitro* growth characteristics and grew to similar endpoint titers in both cell types (Figure 1). ZIKV-Paraiba is more closely related to the ZIKV strains of the Asian-lineage, including ZIKV-French Polynesia (Figure S1), with a whole-genome nucleotide identity of 98.8%. The African lineage is more phylogenetically distinct; ZIKV-Paraiba shows only 88.6% nucleotide identity with ZIKV-MR766 which has a deletion in the E protein in a critical glycosylation site potentially affecting virulence (Haddow et al., 2016). Our results, however, demonstrate that these genetic differences do not significantly affect *in vitro* growth.

Mice lacking interferon responses have been described as ZIKV disease models recapitulating human disease to some degree including the development of meningoencephalitis and fetal brain malformations (Miner and Diamond, 2017; Morrison and Diamond, 2017). Mice lacking both the type I and II interferon responses (e.g., AG129 mice) developed more severe disease and succumbed to ZIKV infection independent of the inoculation route compared to mice lacking only the type I interferon response (e.g., IFNAR^−/−^ mice) (Morrison and Diamond, 2017). We favored the IFNAR^−/−^ mouse model as these mice, despite being immunocompromised, can still develop protective immune responses (Hinkula et al., 2017). In our first mouse study, ZIKV-Paraiba infection of adult IFNAR^−/−^ mice (5–8-weeks old) resulted in similar signs of disease and neuropathological brain changes (e.g., lymphocyte infiltration, neuronal necrosis and perivascular cuffing) as described previously for A129 mice at the same age infected with an African ZIKV strain by the same route (Dowall et al., 2016). In previous studies using footpad or s.c. inoculation, ZIKV-specific RNA levels and titers increased in the brain over time (Dowall et al., 2016; Lazear et al., 2016; Tripathi et al., 2017), similar to what we observed here (Figures 2C,D) validating our model. In our study i.p. infection was the only route resulting in uniform lethality in young IFNAR^−/−^ mice (Figure 4). While i.p. inoculation is not a natural route of infection, it does provide the most consistent model for testing antiviral countermeasures with an LD_50_ for ZIKV-Paraiba of 1 PFU (Figure S2).

For the establishment of a challenge model for vaccine efficacy testing, older mice needed to be evaluated as developing immunity upon vaccination requires time. Other groups have used AG129 mice deficient of type I and II interferon responses to achieve uniform lethality in control/placebo groups despite their limitations in responding to vaccination (Richner et al., 2017). As mentioned above, we chose to test IFNAR^−/−^ mice in our studies as they are still able to develop protective immune responses to infection and vaccination (Hinkula et al., 2017). Young A129 mice (animals lacking the type I interferon response) have been used for vaccine efficacy testing but do not show a uniform phenotype after challenge resulting in the need for higher numbers in order to obtain statistically significant results (Shan et al., 2017). Unfortunately, ZIKV-Paraiba did not cause mortality >20% in older IFNAR^−/−^ mice (10–12-weeks old) independent of the route of inoculation (Figure 4). Therefore, we tested different ZIKV strains at different doses via i.p. inoculation. Of the two additional ZIKV strains we examined, ZIKV-French Polynesia displayed the most uniform onset of disease. Infection with 10^5^ PFU i.p. resulted in uniform lethality, whereas all other groups contained a subset of animals that recovered from infection (Figure 5). Thus, this model seems to be a promising choice for vaccine efficacy studies. Notably, ZIKV-French Polynesia is one of the ZIKV strains used in nonhuman primate (NHP) studies (Aliota et al., 2016b; Dudley et al., 2016; Morrison and Diamond, 2017) and, therefore, appears to be a good choice for vaccine efficacy screening studies in mice prior to efficacy testing in NHPs for licensing purposes.

In summary, we were able to establish uniformly lethal IFNAR^−/−^ mouse models utilizing two distinct ZIKV strains form the Asian lineage that are suitable for efficacy testing of treatment and vaccine approaches.

## Author contributions

AM and HF: designed the studies; AM, JE, JC, KM, NA, SC, and CM: performed the experiments; RR and DPS: processed and analyzed the pathology samples. AM, DS, SW, SB, and HF: analyzed the data; AM and HF: wrote the manuscript. All authors approved the manuscript.

### Conflict of interest statement

The authors declare that the research was conducted in the absence of any commercial or financial relationships that could be construed as a potential conflict of interest.

## References

[B1] Aliota M. T. Caine E. A. Walker E. C. Larkin K. E. Camacho E. Osorio J. E. (2016a). Characterization of lethal zika virus infection in AG129 mice. PLoS Negl. Trop. Dis. 10:e0004682 10.1371/journal.pntd.0004682 27093158 PMC4836712

[B2] Aliota M. T. Dudley D. M. Newman C. M. Mohr E. L. Gellerup D. D. Breitbach M. E.. (2016b). Heterologous protection against asian zika virus challenge in rhesus macaques. PLoS Negl. Trop. Dis. 10:e0005168. 10.1371/journal.pntd.0005168 27911897 PMC5135040

[B3] Bearcroft W. G. (1956). Zika virus infection experimentally induced in a human volunteer. Trans. R. Soc. Trop. Med. Hyg. 50, 442–448. 10.1016/0035-9203(56)90090-6 13380987

[B4] Cao-Lormeau V. M. Blake A. Mons S. Lastere S. Roche C. Vanhomwegen J.. (2016). Guillain-Barre Syndrome outbreak associated with Zika virus infection in French Polynesia: a case-control study. Lancet 387, 1531–1539. 10.1016/S0140-6736(16)00562-6 26948433 PMC5444521

[B5] Dick G. W. (1952). Zika virus. II. Pathogenicity and physical properties. Trans. R. Soc. Trop. Med. Hyg. 46, 521–534. 10.1016/0035-9203(52)90043-6 12995441

[B6] Dick G. W. Kitchen S. F. Haddow A. J. (1952). Zika virus. I. Isolations and serological specificity. Trans. R. Soc. Trop. Med. Hyg. 46, 509–520. 10.1016/0035-9203(52)90042-4 12995440

[B7] Dowall S. D. Graham V. A. Rayner E. Atkinson B. Hall G. Watson R. J.. (2016). A susceptible mouse model for zika virus infection. PLoS Negl. Trop. Dis. 10:e0004658. 10.1371/journal.pntd.0004658 27149521 PMC4858159

[B8] Dowd K. A. DeMaso C. R. Pelc R. S. Speer S. D. Smith A. R. Y. Goo L.. (2016). Broadly neutralizing activity of Zika Virus-immune sera identifies a single viral serotype. Cell Rep. 16, 1485–1491. 10.1016/j.celrep.2016.07.049 27481466 PMC5004740

[B9] Dudley D. M. Aliota M. T. Mohr E. L. Weiler A. M. Lehrer-Brey G. Weisgrau K. L.. (2016). A rhesus macaque model of Asian-lineage Zika virus infection. Nat. Commun. 7:12204. 10.1038/ncomms12204 27352279 PMC4931337

[B10] Duffy M. R. Chen T. H. Hancock W. T. Powers A. M. Kool J. L. Lanciotti R. S.. (2009). Zika virus outbreak on Yap Island, Federated States of Micronesia. N. Engl. J. Med. 360, 2536–2543. 10.1056/NEJMoa0805715 19516034

[B11] Faye O. Faye O. Diallo D. Diallo M. Weidmann M. Sall A. A. (2013). Quantitative real-time PCR detection of Zika virus and evaluation with field-caught mosquitoes. Virol. J. 10:311. 10.1186/1743-422X-10-311 24148652 PMC4016539

[B12] Faye O. Freire C. C. Iamarino A. Faye O. de Oliveira J. V. Diallo M.. (2014). Molecular evolution of Zika virus during its emergence in the 20(th) century. PLoS Negl. Trop. Dis. 8:e2636. 10.1371/journal.pntd.0002636 24421913 PMC3888466

[B13] Fonseca K. Meatherall B. Zarra D. Drebot M. MacDonald J. Pabbaraju K.. (2014). First case of Zika virus infection in a returning Canadian traveler. Am. J. Trop. Med. Hyg. 91, 1035–1038. 10.4269/ajtmh.14-0151 25294619 PMC4228871

[B14] Frank C. Cadar D. Schlaphof A. Neddersen N. Günther S. Schmidt-Chanasit J.. (2016). Sexual transmission of Zika virus in Germany, April 2016. Euro. Surveill. 21:30252. 10.2807/1560-7917.ES.2016.21.23.30252 27311329

[B15] Haddow A. D. Nasar F. Guzman H. Ponlawat A. Jarman R. G. Tesh R. B.. (2016). Genetic characterization of Spondweni and Zika Viruses and susceptibility of geographically distinct strains of aedes aegypti, aedes albopictus and culex quinquefasciatus (*Diptera*: Culicidae) to Spondweni Virus. PLoS Negl. Trop. Dis. 10:e0005083. 10.1371/journal.pntd.0005083 27783682 PMC5082648

[B16] Hayes E. B. (2009). Zika virus outside Africa. Emerg. Infect. Dis. 15, 1347–1350. 10.3201/eid1509.090442 19788800 PMC2819875

[B17] Hinkula J. Devignot S. Åkerström S. Karlberg H. Wattrang E. Bereczky S.. (2017). Immunization with DNA plasmids coding for crimean-congo hemorrhagic fever virus capsid and envelope proteins and/or virus-like particles induces protection and survival in challenged mice. J. Virol. 91:e02076–16. 10.1128/JVI.02076-16 28250124 PMC5411611

[B18] Katoh K. Misawa K. Kuma K. Miyata T. (2002). MAFFT: a novel method for rapid multiple sequence alignment based on fast Fourier transform. Nucleic Acids Res. 30, 3059–3066. 10.1093/nar/gkf436 12136088 PMC135756

[B19] Kostyuchenko V. A. Lim E. X. Zhang S. Fibriansah G. Ng T. S. Ooi J. S.. (2016). Structure of the thermally stable Zika virus. Nature 533, 425–428. 10.1038/nature17994 27093288

[B20] Kuno G. Chang G. J. (2007). Full-length sequencing and genomic characterization of Bagaza, Kedougou, and Zika viruses. Arch. Virol. 152, 687–696. 10.1007/s00705-006-0903-z 17195954

[B21] Lazear H. M. Govero J. Smith A. M. Platt D. J. Fernandez E. Miner J. J.. (2016). A mouse model of Zika Virus pathogenesis. Cell Host Microbe 19, 720–730. 10.1016/j.chom.2016.03.010 27066744 PMC4866885

[B22] Liu Y. Liu J. Du S. Shan C. Nie K. Zhang R.. (2017). Evolutionary enhancement of Zika virus infectivity in Aedes aegypti mosquitoes. Nature 545, 482–486. 10.1038/nature22365 28514450 PMC5885636

[B23] Miner J. J. Diamond M. S. (2017). Zika Virus pathogenesis and tissue tropism. Cell Host Microbe 21, 134–142. 10.1016/j.chom.2017.01.004 28182948 PMC5328190

[B24] Miner J. J. Sene A. Richner J. M. Smith A. M. Santeford A. Ban N.. (2016). Zika virus infection in mice causes panuveitis with shedding of virus in tears. Cell Rep. 16, 3208–3218. 10.1016/j.celrep.2016.08.079 27612415 PMC5040391

[B25] Mlakar J. Korva M. Tul N. Popovic M. Poljsak-Prijatelj M. Mraz J.. (2016). Zika Virus Associated with Microcephaly. N. Engl. J. Med. 374, 951–958. 10.1056/NEJMoa1600651 26862926

[B26] Morrison T. E. Diamond M. S. (2017). Animal models of Zika Virus infection, pathogenesis, and immunity. J. Virol. 91:e00009–17. 10.1128/JVI.00009-17 28148798 PMC5375682

[B27] Musso D. Roche C. Robin E. Nhan T. Teissier A. Cao-Lormeau V. M. (2015). Potential sexual transmission of Zika virus. Emerg. Infect. Dis. 21, 359–361. 10.3201/eid2102.141363 25625872 PMC4313657

[B28] Reed L. J. Muench H. (1938). A simple method of estimating fifty percent endpoints. Am. J. Hyg. 27, 493–497.

[B29] Richner J. M. Himansu S. Dowd K. A. Butler S. L. Salazar V. Fox J. M.. (2017). Modified mRNA vaccines protect against Zika Virus infection. Cell 169, 176. 10.1016/j.cell.2017.03.016 28340344

[B30] Rossi S. L. Tesh R. B. Azar S. R. Muruato A. E. Hanley K. A. Auguste A. J.. (2016). Characterization of a Novel Murine Model to Study Zika Virus. Am. J. Trop. Med. Hyg. 94, 1362–1369. 10.4269/ajtmh.16-0111 27022155 PMC4889758

[B31] Shan C. Muruato A. E. Nunes B. T. D. Luo H. Xie X. Medeiros D. B. A.. (2017). A live-attenuated Zika virus vaccine candidate induces sterilizing immunity in mouse models. Nat. Med. 23, 763–767. 10.1038/nm.4322 28394328 PMC6276361

[B32] Simpson D. I. (1964). Zika Virus infection in man. Trans. R. Soc. Trop. Med. Hyg. 58, 335–338. 10.1016/0035-9203(64)90201-9 14175744

[B33] Tripathi S. Balasubramaniam V. R. Brown J. A. Mena I. Grant A. Bardina S. V.. (2017). A novel Zika virus mouse model reveals strain specific differences in virus pathogenesis and host inflammatory immune responses. PLoS Pathog. 13:e1006258. 10.1371/journal.ppat.1006258 28278235 PMC5373643

[B34] Tsetsarkin K. A. Kenney H. Chen R. Liu G. Manukyan H. Whitehead S. S.. (2016). A full-length infectious cDNA clone of Zika Virus from the 2015 Epidemic in Brazil as a Genetic Platform for Studies of Virus-Host Interactions and vaccine development. MBio 7:e01114–16. 10.1128/mBio.01114-16 27555311 PMC4999549

[B35] Wang F. Flanagan J. Su N. Wang L. C. Bui S. Nielson A.. (2012). RNAscope: a novel *in situ* RNA analysis platform for formalin-fixed, paraffin-embedded tissues. J. Mol. Diagn. 14, 22–29. 10.1016/j.jmoldx.2011.08.002 22166544 PMC3338343

[B36] Zanluca C. Melo V. C. Mosimann A. L. Santos G. I. Santos C. N. Luz K. (2015). First report of autochthonous transmission of Zika virus in Brazil. Mem. Inst. Oswaldo Cruz 110, 569–572. 10.1590/0074-02760150192 26061233 PMC4501423

