# Supplementary material for: Lethal Zika Virus Disease Models in Young and Older Interferon α/β Receptor Knock Out Mice
Source: Front Cell Infect Microbiol. 2018 Apr 11;8:117. doi: 10.3389/fcimb.2018.00117 (PMC5904210; doi:10.3389/fcimb.2018.00117)
Supplement: Supplementary file 1 [file Image1.PDF]

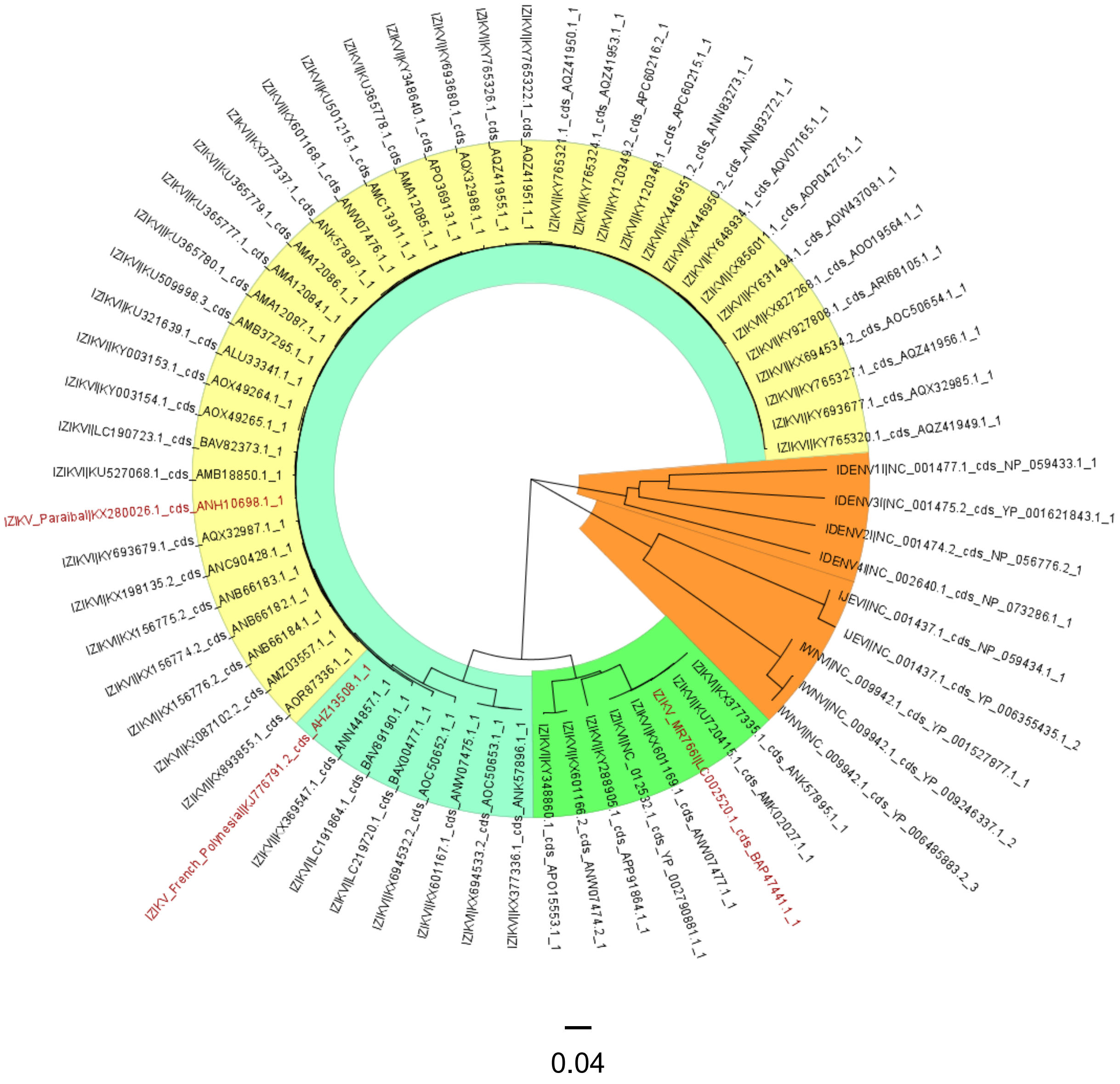

Figure S1: Circular tree depicting the phylogenetic relationship of 65 flavivirus polyprotein coding regions. The clade highlighted in orange includes Dengue virus (DENV), Japanese Encephalitis virus (JEV), and West Nile virus (WNV). The clade highlighted in green includes ZIKV sequences of the African lineage. The clade highlighted in cyan includes ZIKV sequences of the Asian lineage. ZIKV sequences of the American sublineage are highlighted in yellow. The three sequences of interest for this study are indicated in red.

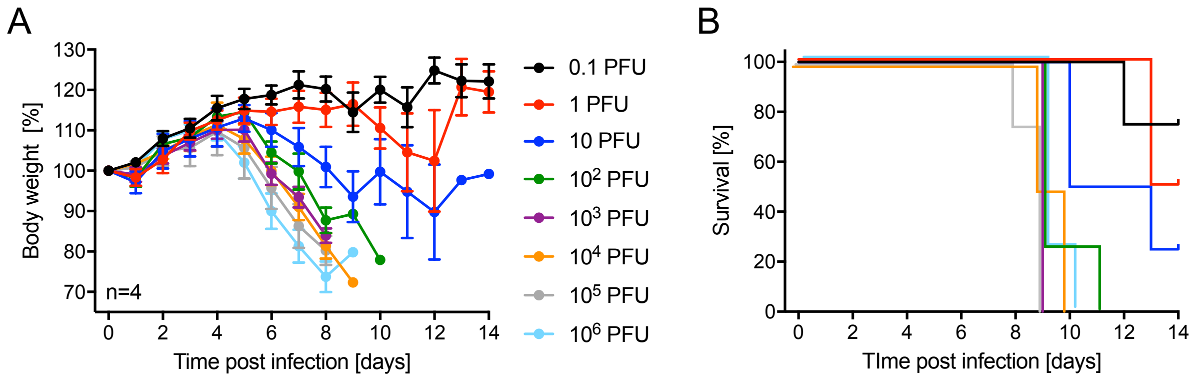

Figure S2: ZIKV-Paraiba dose evaluation in young mice. Groups of four mice (2 male, 2 female, 4 weeks old) were intraperitoneally infected with the indicated doses of ZIKV-Paraiba. (A) Body weight changes and (B) survival curves are shown. Error bars indicate standard deviation.

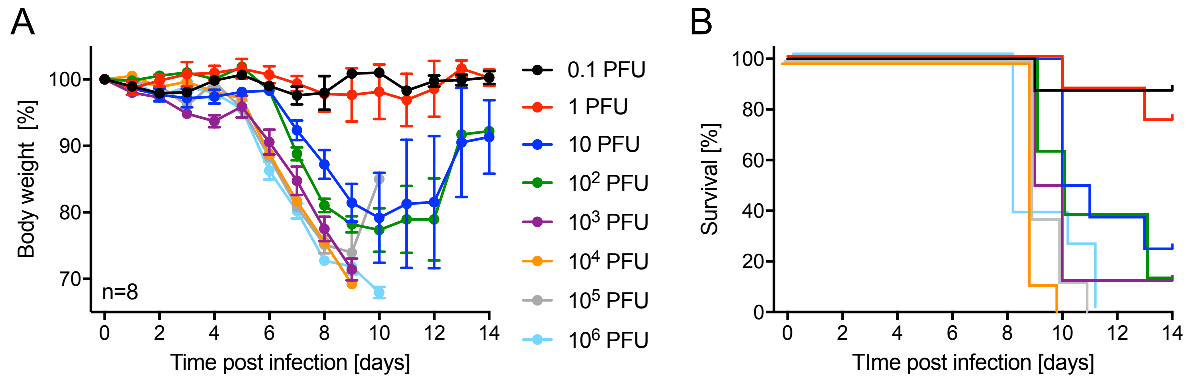

Figure S3: ZIKV-French Polynesia dose evaluation in older mice. Groups of eight mice (4 male, 4 female, 10-12 weeks old) were intraperitoneally infected with the indicated doses of ZIKV-French Polynesia. (A) Body weight changes and (B) survival curves are shown. Error bars indicate standard deviation.
